# A Comparative Study of Blood Loss With and Without Infusion of Tranexamic Acid in Total Knee Replacement

**DOI:** 10.7759/cureus.27737

**Published:** 2022-08-06

**Authors:** Divyansh Kumar, Anshu Sharma, Gaurav Sharma, Aalap Trivedi

**Affiliations:** 1 Orthopaedics, Sawai Man Singh Medical College, Jaipur, IND; 2 Orthopaedics, Geetanjali Medical College and Hospital, Udaipur, IND; 3 Internal Medicine, Geetanjali Medical College and Hospital, Udaipur, IND; 4 Orthopaedics, M. P. Shah Government Medical College and Hospital, Jamnagar, IND

**Keywords:** total knee replacement, tranexamic acid, blood transfusion, hemoglobin, perioperative blood loss, total knee arthroplasty

## Abstract

Introduction: Total knee replacement (TKR) is associated with significant blood loss in intra- and postoperative periods. This trial was designed to determine the efficacy of tranexamic acid (TXA) in the reduction of perioperative blood loss and the need for blood transfusion in patients undergoing primary TKR.

Materials and methods: This study was performed at a tertiary care institute with 30 cases of primary unilateral TKR. The patients were randomly divided into two groups comprising 15 patients each. Group A comprised patients who received TXA by intravenous route and locally. Group B served as control, which comprised patients who had not received TXA. Patients were assessed in terms of intraoperative and postoperative blood loss, reduction in haemoglobin, the requirement of blood transfusion, and any untoward effects of TXA at 24 and 72 hours after surgery.

Results: In group A, the mean number of swabs used intraoperatively was 2.3 ± 0.5 swabs while in group B, the mean number was 4.3 ± 0.7 swabs (p = 0.0000). The mean drop in the postoperative haemoglobin concentration in group A was 0.6 gm/dl (24 hours) and 1.3 gm/dl (72 hours), with a mean postoperative drain collection of 247.3 ± 50.6 ml (24 hours) and 316.7 ± 55.6 ml (72 hours). In comparison, the mean drop in the postoperative haemoglobin in group B was 1.5 gm/dl (24 hours) and 2.3 gm/dl (72 hours), with a mean drain collection of 474 ± 30.7 ml (24 hours) and 453.3 ± 37.7 ml (72 hours) (p < 0.001). In group A, significantly fewer patients (four) required blood transfusion while 13 patients required blood transfusion in group B (p = 0.0004).

Conclusion: The data from this study conclude that the use of TXA in TKR significantly reduces perioperative blood loss and the need for postoperative blood transfusion without significantly altering the liver and renal functions and coagulation profile of patients.

## Introduction

Arthritic joint failure is a common and crippling disease causing severe pain and significant disability. Although medical treatments such as pain killers and local injections can be successful in the early stages, they are less effective in the advanced stages of the disease. In 1972, Dr John Insall designed what has become the prototype for current total knee replacements (TKRs). It was called the total condylar knee. This was a prosthesis made of three components, which would resurface all three surfaces of the knee: the femur, tibia, and patella. They were each fixed with bone cement and the results were significant [1]. Today, TKR has become one of the most common operations in orthopaedic practice. TKR significantly reduces pain and enhances the quality of life [2].

However, there are some risks associated with TKR surgery. The potential for bleeding and the need for transfusions should be noted. There has been evidence that TKR surgery results in considerable blood loss and occasionally necessitates blood transfusions [3-5]. A patient who is undergoing TKR can lose up to 2300 ml of blood, which is almost one-third of the total circulating blood volume [6]. Bleeding during total knee arthroplasty can be from different factors such as patient characteristics (anticoagulation, cirrhosis, haemophiliac, etc.) and surgical technique (bone cuts and soft tissue dissection). In some studies, the transfusion rate after TKR has been as high as 30% [5]. The method of transfusing blood products is not without risk; these risks include infection, severe systemic reactions, and even death [7]. Transfusions also increase rehabilitation time and lengthen hospital stay and the cost of treatment [8,9]. Controlling blood loss during and after surgery is thus a key objective to TKR success. One such method is to use tranexamic acid (TXA) during TKR surgery. We conducted a prospective case-control study to determine the efficacy of TXA in the reduction of perioperative and postoperative blood loss in TKR, to determine the reduction in the need for allogenic blood transfusion in patients undergoing TKR, and to know any untoward effects of TXA on the liver and renal functions and coagulation profile.

## Materials and methods

This study was performed at a tertiary care medical college centre after obtaining institutional ethics committee clearance (Institutional Ethics Committee, Mahatma Gandhi Medical College and Hospital, Jaipur, Rajasthan: MGMCH/IEC/JPR/2014/203) and prior written informed consent from the patients. The study included all cases of primary unilateral TKR done in the orthopaedic department during the study period irrespective of age, sex, type of prosthesis, and technique used for surgery.

Cases of revision TKR, patients with a known history of a bleeding disorder, deep vein thrombosis (DVT), pulmonary embolism, or anticoagulant therapy were excluded from the study. Cases undergoing TKR for trauma or tumour and patients with a history of renal failure and liver disorder were also excluded.

Patients fulfilling the above criteria were included in this prospective case-control study. The patients were randomly divided into two groups comprising 15 patients each. Group A included patients who had undergone primary TKR and who received 1 gram of TXA intravenously at the time of induction of anaesthesia, again 1 gram TXA injection given intravenously before deflation of tourniquet, and 1 gram TXA sprayed locally in the knee before closure. Group B included patients who had undergone primary TKR in which TXA was not used.

Surgeries were performed in a standard manner. All the patients in both groups had been operated on by anterior mid-line incision and fixed bearing metal back implants were used. A pressurised wash was given with 500 ml of normal saline in both groups. Closure of wound over drain and release of the tourniquet was done in a standard manner in both groups. Blood loss was calculated during the operation by counting the number of swabs soaked during surgery and in the postoperative period by measuring drain collection at 24-hour and 72-hour intervals, before removal of the drain in both groups.

Haemoglobin, serum glutamic-oxaloacetic transaminase (SGOT), serum glutamic pyruvic transaminase (SGPT), creatinine, urea, and coagulation profiles like bleeding time (BT), clotting time (CT), prothrombin time (PT), and international normalized ratio (INR) were measured preoperatively and at 24 and 72 hours postoperatively in both groups.

The Student’s t-test or χ2 test was used to evaluate the differences between the groups. P-value < 0.05 was used as the cut-off for statistically significant differences.

## Results

Age

In group A, the minimum age of the patients was 50 years and the maximum age was 80 years (mean: 61.9 ± 9 years). In group B, the minimum age of the patients was 50 years and the maximum was 72 years (mean: 63.1 ± 5.7 years). There was no significant difference in age distribution in both the groups (p = 0.6660) (Table 1).

**Table 1:** Age distribution The mean age in group A was 61.9 ± 9 years, and in group B, it was 63.1 ± 5.7 years. There was no significant difference in age distribution in both groups (p = 0.6660).

Age	Group A		Group B	
	No.	%	No.	%
50-59	6	40.0	2	13.3
60-69	7	46.7	10	66.7
70-80	2	13.3	3	20.0
Total	15	100.0	15	100.0

Gender

Group A consisted of nine females (60%) while group B consisted of eight females (53.3%). Group A consisted of six males (40%) and group B had seven (46.7%) males. Both groups had no significant difference in demographic profile (p > 0.05) (Table 2).

**Table 2:** Gender distribution Both the groups had no significant difference in gender distribution (p > 0.05).

Sex	Group A		Group B	
	No.	%	No.	%
Female	9	60.0	8	53.3
Male	6	40.0	7	46.7
Total	15	100.0	15	100.0

Site

In group A, the number of left-sided knees operated was eight (53.3%), and in group B, it was nine (60%). While right side knees in group A were seven (46.7%), in group B, right side knees were six (40%). Statistically, both groups had no significant difference in the side of the knee operated (p > 0.05) (Table 3).

**Table 3:** Site Both groups had no statistically significant difference in the side of the knee operated (p > 0.05).

Site	Group A		Group B	
	No.	%	No.	%
Left	8	53.3	9	60.0
Right	7	46.7	6	40.0
Total	15	100.0	15	100.0

Number of swabs used intraoperatively

In group A, the minimum number of swabs (standard size 25 x 25 cm) used during surgery was two and the maximum was three (mean: 2.3 ± 0.5 swabs), while in group B, the minimum number of swabs used during surgery was three and the maximum was five (mean: 4.3 ± 0.7 swabs). In group A, in 66.7% of cases, only two swabs were used, while in group B, four swabs were used in 46.7% of cases and five swabs were used in 40% of cases during surgery. In group A, statistically significant fewer swabs were used during surgery (p = 0.0000), which means the use of TXA significantly reduces intraoperative blood loss (Table 4).

**Table 4:** Number of swabs used intraoperatively The mean number of swabs used during surgery in group A was 2.3 ± 0.5 swabs, while in group B, it was 4.3 ± 0.7 swabs. In group A, statistically significant fewer swabs were used during surgery (p = 0.0000).

No. of sponges soaked	Group A		Group B				
	No. of patients	%	No. of patients	%			
2	10	66.7					
3	5	33.3	2	13.3			
4			7	46.7			
5			6	40.0			
Total	15	100.0	15	100.0			
Soakage	Group A			Group B			P-value b/w groups
	Mean	SD	P-value	Mean	SD	P-value	
	2.3	0.5		4.3	0.7		0.0000

Drain collection

In group A, the minimum postoperative drain at 24 hours was 150 ml and the maximum was 300 ml (mean: 247.3 ± 50.6 ml). In group B, the minimum postoperative drain at 24 hours was 400 ml and the maximum was 500 ml (mean: 474 ± 30.7 ml). In group A, eight patients (53.3%) had a postoperative drain at 24 hours between 200 and 299 ml and six patients (40%) had between 300 and 399 ml, while in group B, all 15 patients (100%) had drain between 400 and 500 ml, which shows that group A had statistically significant less drain collection 24 hours postoperatively (p = 0.0000).

In group A, the minimum postoperative drain at 72 hours was 200 ml and the maximum was 400 ml (mean: 316.7 ± 55.6 ml). In group B, the minimum postoperative drain at 72 hours was 400 ml and the maximum was 500 ml (mean: 453.3 ± 37.7 ml). In group A, 10 patients (66.7%) had a postoperative drain at 72 hours between 300 and 399 ml, while in group B, all 15 patients (100%) had a drain between 400 and 500 ml, which shows that group A had statistically significant less drain collection 72 hours postoperatively (p = 0.0000). This shows that TXA significantly reduces postoperative bleeding in TKR patients (Table 5).

**Table 5:** Drain collection Group A had statistically significant less drain collection at 24 hours and 72 hours postoperatively (p = 0.0000).

Drain (ml)	Group A				Group B			
	Postoperative 24 hours		Postoperative 72 hours		Postoperative 24 hours		Postoperative 72 hours	
	No.	%	No.	%	No.	%	No.	%
<200	1	6.7		0.0		0.0		0.0
200-299	8	53.3	3	20.0		0.0		0.0
300-399	6	40.0	10	66.7		0.0		0.0
400-500		0.0	2	13.3	15	100.0	15	100.0
Total	15	100.0	15	100.0	15	100.0	15	100.0

	Group A			Group B			P-value b/w groups
Drain	Mean	SD	P-value	Mean	SD	P-value	
24 hours	247.3	50.6		474.0	30.7		0.0000
72 hours	316.7	55.6		453.3	37.4		0.0000

Haemoglobin

In group A, the minimum preoperative haemoglobin was 10.9 gm/dl and the maximum was 15.2 gm/dl (mean: 12.9 ± 1.2 gm/dl). In group B, the minimum preoperative haemoglobin was 10.4 gm/dl and the maximum was 15.2 gm/dl (mean: 12.6 ± 1.6 gm/dl). Both groups had 33.3% of patients with haemoglobin in the 12-13 and 13-14 gm/dl range. Both groups had no significant difference in the level of preoperative haemoglobin distribution (p > 0.05).

In group A, the minimum postoperative haemoglobin at 24 hours was 10 gm/dl and the maximum was 14.9 gm/dl (mean: 12.4 ± 1.4 gm/dl). In group B, the minimum postoperative haemoglobin at 24 hours was 9.8 gm/dl and the maximum was 11.8 gm/dl (mean: 10.5 ± 0.6 gm/dl), which shows a statistically significant fall in postoperative haemoglobin at 24 hours in group B (p = 0.0015).

In group A, the minimum postoperative haemoglobin at 72 hours was 9.5 gm/dl and the maximum was 13.9 gm/dl (mean: 11.7 ± 1.4 gm/dl). In group B, the minimum postoperative haemoglobin at 72 hours was 8.9 gm/dl and the maximum was 11 gm/dl (mean: 9.6 ± 0.7 gm/dl). In group A, 80% of patients had haemoglobin > 10 gm/dl at 72 hours postoperatively, while in group B, only 33.4% had haemoglobin > 10 gm/dl at 72 hours postoperatively, which shows a statistically significant fall in postoperative haemoglobin at 72 hours in group B (p = 0.0001) (Table 6).

**Table 6:** Haemoglobin Group B shows a statistically significant fall in postoperative haemoglobin at 24 hours and at 72 hours (p = 0.0015).

Haemoglobin	Group A						Group B					
	Preoperative		Postoperative 24 hours		Postoperative 72 hours		Preoperative		Postoperative 24 hours		Postoperative 72 hours	
	No.	%	No.	%	No.	%	No.	%	No.	%	No.	%
<10					3	20.0					10	66.7
10-11	1	6.7	3	20.0	3	20.0	2	13.3	3	20.0	4	26.7
11-12	2	13.3	3	20.0	2	13.3	1	6.7	9	60.0	1	6.7
12-13	5	33.3	3	20.0	5	33.3	5	33.3	3	20.0		
13-14	5	33.3	4	26.7	2	13.3	5	33.3		0.0		
14-15	1	6.7	2	13.3			1	6.7		0.0		
15-16	1	6.7					1	6.7				
Total	15	100.0	15	100.0	15	100.0	15	100.0	15	100.0	15	100.0

	Group A			Group B			P-value b/w groups
	Mean	SD	P-value	Mean	SD	P-value	
Preoperative	12.9	1.2		12.6	1.6		0.5538
Postoperative 24 hours	12.4	1.4	0.0015	10.5	0.6	0.0000	0.0001
Postoperative 72 hours	11.7	1.4	0.0001	9.6	0.7	0.0000	0.0001

Fall in haemoglobin

In group A, the fall of haemoglobin at 24 hours was 0.6 ± 0.4 mg/dl, and in group B, it was 1.5 ± 1.1 mg/dl. The fall of haemoglobin at 72 hours in group A was 1.3 ± 0.8 mg/dl, and in group B, it was 2.3 ± 1.3 mg/dl, which shows significantly less haemoglobin reduction in group A (p = 0.0001) (Table 7).

**Table 7:** Fall of haemoglobin Group A shows significantly less haemoglobin reduction (p = 0.0001).

		Fall of haemoglobin at 24 hours	Fall of haemoglobin at 72 hours
Group A			
	Mean	0.6	1.3
	SD	0.4	0.8
Group B			
	Mean	1.5	2.3
	SD	1.1	1.3
P-value b/w groups		0.0001	0.0001

Postoperative blood transfusion

Postoperatively in group A, only four patients (26.7%) needed a blood transfusion, while in group B, 13 patients (86.7%) needed a blood transfusion; this shows that a significantly less number of patients required postoperative blood transfusion in group A (p = 0.0004) (Table 8).

**Table 8:** Postoperative blood transfusion In group A, a significantly less number of patients required postoperative blood transfusion (p = 0.0004).

Postoperative blood transfusion	Group A		Group B	
	No.	%	No.	%
No	11	73.3	2	13.3
Yes	4	26.7	13	86.7
Total	15	100.0	15	100.0

Liver functions (SGOT)

In group A, the minimum preoperative SGOT was 25 U/L and the maximum was 49 U/L (mean: 33.5 ± 8.1 U/L), and in a maximum of 46.7% of patients, SGOT was in the range of 25-29 U/L. While in group B, the minimum preoperative SGOT was 16 U/L and the maximum was 49 U/L (mean: 32.4 ± 8.9 U/L), and in a maximum of 46.7% of patients, SGOT was in the range of 25-29 U/L, which shows no statistically significant difference in preoperative SGOT in both groups (p = 0.7166).

In group A, the minimum postoperative SGOT at 24 hours was 25 U/L and the maximum was 49 U/L (mean: 33.6 ± 8.1 U/L), and in maximum patients (46.7%), SGOT was in the range of 25-29 U/L. While in group B, the minimum postoperative SGOT at 24 hours was 22 U/L and the maximum was 49 U/L (mean: 32.5 ± 9 U/L), and in maximum patients (46.7%), SGOT was in the range of 25-29 U/L, which shows no statistically significant difference in postoperative SGOT at 24 hours in both groups (p = 0.7784).

In group A, the minimum postoperative SGOT at 72 hours was 25 U/L and the maximum was 49 U/L (mean: 32.8 ± 7.8 U/L), and in maximum patients (46.7%), SGOT was in the range of 25-29 U/L. While in group B, the minimum postoperative SGOT at 72 hours was 22 U/L and the maximum was 49 U/L (mean: 32.4 ± 8.9 U/L), and in maximum patients (46.7%), SGOT was in the range of 25-29 U/L, which shows no statistically significant difference in postoperative SGOT at 72 hours in both groups (p = 0.357) (Table 9).

**Table 9:** Effect of tranexamic acid on liver functions (SGOT) Tranexamic acid does not affect SGOT values in 24 and 72 hours postoperative reports. SGOT: serum glutamic-oxaloacetic transaminase.

SGOT	Group A			Group B		
	Preoperative	Postoperative 24 hours	Postoperative 72 hours	Preoperative	Postoperative 24 hours	Postoperative 72 hours
20-24				13.3%	13.3%	13.3%
25-29	46.7%	46.7%	46.7%	46.7%	46.7%	46.7%
30-34	20.0%	20.0%	20.0%	6.7%	6.7%	6.7%
35-39	13.3%	13.3%	20.0%	13.3%	13.3%	13.3%
40-44	6.7%	6.7%				6.7%
45-49	13.3%	13.3%	13.3%	20.0%	20.0%	13.3%
Grand total	100.0%	100.0%	100.0%	100.0%	100.0%	100.0%

Liver functions (SGPT)

In group A, the minimum preoperative SGPT was 16 U/L and the maximum was 42 U/L (mean: 27.4 ± 8.1 U/L), and in maximum patients (73.3%), SGPT was in the range of 20-29 U/L. While in group B, the minimum preoperative SGPT was 16 U/L and the maximum was 42 U/L (mean: 26.5 ± 6.5 U/L), and in maximum patients (73.3%), SGPT was in the range of 25-29 U/L, which shows no statistically significant difference in preoperative SGPT in both groups (p = 0.6865).

In group A, the minimum SGPT postoperatively at 24 hours was 17 U/L and the maximum was 43 U/L (mean: 27.8 ± 5.9 U/L), and in maximum patients (73.3%), SGPT was in the range of 20-29 U/L. While in group B, the minimum SGPT postoperatively at 24 hours was 17 U/L and the maximum was 43 U/L (mean: 27.7 ± 6.6 U/L), and in maximum patients (73.3%), SGPT was in the range of 25-29 U/L, which shows no statistically significant difference in SGPT postoperatively at 24 hours in both groups (p = 0.9710).

In group A, the minimum SGPT postoperatively at 72 hours was 16 U/L and the maximum was 42 U/L (mean: 27.9 ± 5.9 U/L), and in maximum patients (60%), SGPT was in the range of 20-29 U/L. While in group B, the minimum SGPT postoperatively at 72 hours was 16 U/L and the maximum was 42 U/L (mean: 26.5 ± 6.5 U/L), and in maximum patients (66.7%), SGPT was in the range of 25-29 U/L, which shows no statistically significant difference in SGPT postoperatively at 72 hours in both groups (p = 0.5420) (Table 10).

**Table 10:** Effect of tranexamic acid on liver functions (SGPT) Tranexamic acid does not affect SGPT values in 24 and 72 hours postoperatively. SGPT: serum glutamic pyruvic transaminase.

SGPT	Group A			Group B		
	Preoperative	Postoperative 24 hours	Postoperative 72 hours	Preoperative	Postoperative 24 hours	Postoperative 72 hours
<20	6.7%	6.7%	6.7%	13.3%	6.7%	13.3%
20-29	73.3%	73.3%	60.0%	66.7%	73.3%	66.7%
30-39	13.3%	13.3%	26.7%	13.3%	13.3%	13.3%
40-49	6.7%	6.7%	6.7%	6.7%	6.7%	6.7%
Grand total	100.0%	100.0%	100.0%	100.0%	100.0%	100.0%

Renal functions

Both the groups had no statistically significant difference in mean values of blood urea and serum creatinine levels preoperatively, at 24 hours postoperatively, and at 72 hours postoperatively (p > 0.05). This shows that TXA does not affect renal functions (Table 11).

**Table 11:** Effect of tranexamic acid on renal functions Tranexamic acid does not affect serum creatinine and blood urea values in 24 and 72 hours postoperatively.

Renal function		Group A	Group B
		Mean	Mean
Preoperative	Blood urea	33.1	33.0
	Serum creatinine	1.0	1.0
Postoperative 24 hours	Blood urea (MG%)	33.1	33.0
	Serum creatinine	1.0	1.1
Postoperative 72 hours	Blood urea (MG%)	33.0	33.0
	Serum creatinine	0.9	1.0

Coagulation profile

Both the groups had no statistically significant difference in mean values of bleeding time, clotting time, and prothrombin time (PT)/international normalized ratio (INR) preoperatively, at 24 hours postoperatively, and at 72 hours postoperatively (p > 0.05). This shows that TXA does not affect the coagulation profile (Table 12).

**Table 12:** Effect of tranexamic acid on coagulation profile Tranexamic acid does not affect the coagulation profile in 24 and 72 hours postoperatively. PT: prothrombin time; INR: international normalized ratio; BT: bleeding time; CT: clotting time.

Bleeding time/clotting time		Group A	Group B
		Mean	Mean
Preoperative	BT	2.1	2.1
	CT	5.2	5.2
Postoperative 24 hours	BT	2.1	2.1
	CT	5.2	5.2
Postoperative 72 hours	BT	2.0	2.1
	CT	5.2	5.2

PT/INR		Group A	Group B
		Mean	Mean
Preoperative	PT	15.6	15.4
	INR	1.2	1.2
Postoperative 24 hours	PT	15.6	15.3
	INR	1.2	1.2
Postoperative 72 hours	PT	15.8	15.4
	INR	1.2	1.2

No complications occurred in this study, specially DVT or any other thrombotic event.

## Discussion

TKR is a frequently done procedure in the modern-day practice of orthopaedics. Limiting blood loss both intraoperatively and postoperatively presents a challenge to the surgeon.

TXA is a lysine analogue. It acts by inhibiting plasminogen activation. TXA competitively inhibits the conversion of plasminogen to plasmin and allows mature fibrin clots to be maintained and coagulation to continue uninhibited. The structure of TXA is composed of C8H15NO2. TXA is solid at room temperature but is completely soluble in water. It has a brief half-life of only two to three hours after ingestion or intravenous injection, and it is swiftly eliminated from the body through the kidneys.

TXA has been available for more than 20 years, with its medical uses ranging from dental extractions, tonsillectomy, cardiac surgery, prostate surgery, menstrual bleeding control, and treatment for patients with haemophilia [10-12]. Furthermore, TXA has been extensively studied in trauma patients and other major surgical sub-specialities (such as thoracic surgery) to decrease blood loss and mortality.

TXA is frequently given to adults in the form of a loading dosage of 10 mg/kg, followed by an infusion of 1 mg/kg/hour [13]. Studies on the use of antifibrinolytics during cardiac surgery served as the main foundation for the values [13]. TXA has been demonstrated in those studies to be beneficial in lowering blood loss and transfusion without posing appreciable concerns for increased mortality, stroke, myocardial infarction, or renal failure [13,14]. In orthopaedics, TXA has recently been gaining favour due to its efficacy and ease of use, both in IV and topical usage. Cost, bio-availability, efficacy, and low complications have helped to increase the common use of TXA in TKR [15,16].

In patients undergoing TKR, intravenously administered TXA (typically 10 mg/kg followed by an infusion of 1 mg/kg/hour) resulted in reductions in postoperative blood losses of 29-54% compared to placebo, with statistically significant decreases in the need for transfusion in some studies. TXA had similar efficacy to aprotinin and was superior to dipyridamole in the reduction of postoperative blood losses [12].

The total number of patients in our study was 30. We randomized into two groups of 15 each; in group A, TXA was given, and in group B, TXA was not given.

In group A, the minimum age was of 50 years and the maximum age was of 80 years (mean: 61.9 ± 9 years). In group B, the minimum age was of 50 years and the maximum age was 72 years (mean: 63.1 ± 5.7 years). Group A consisted of nine females (60%) while group B consisted of eight females (53.3%). While group A consisted of six males (40%) and group B had seven (46.7%). Both the groups had no significant difference in demographic profile (p > 0.05) (Tables 1, 2).

In group A, the number of left-sided knees operated was eight (53.3%) and in group B, it was nine (60%). While right-sided knees in group A were seven (46.7%) and in group B were six (40%); statistically, both groups had no significant difference in the form of sites operated (p > 0.05) (Table 3).

In our study, intraoperative blood loss was measured as the mean number of swabs used during surgery. It was 2.3 swabs in group A and 4.3 swabs in group B, which shows that there was significantly less intraoperative blood loss in the TXA group (p = 0.0000), which is consistent with a study done by Yamasaki et al. [17] and Yang et al. [18] (Table 4).

In our study, postoperative collection in drain at 24 hours in group A was 247.30 ml, and in group B, it was 447.30 ml, and at 72 hours, it was 316.70 ml in group A and 453.30 ml in group B, which shows that TXA significantly reduces postoperative blood loss in drain (Table 5). Results are similar to a study done by Kelley et al. [19], which shows a significant reduction in postoperative drain collection in the TXA group.

In our study, the mean postoperative haemoglobin in group A was 12.4 ± 1.4 gm/dl (24 hours) and 11.7 ± 1.4 gm/dl (72 hours), and in group B, it was 10.5 ± 0.6 gm/dl (24 hours) and 9.6 ± 0.7 gm/dl (72 hours), which shows a significant decrease in haemoglobin in the non-TXA group (Table 6). These findings are consistent with a study conducted by Kelley et al. [19].

In this study, postoperative fall of haemoglobin at 24 hours in group A was 0.6 ± 0.4 mg/dl and 1.5 ± 1.1 mg/dl in group B, and at 72 hours, it was 1.3 ± 0.8 mg/dl in group A and 2.3 ± 1.3 mg/dl in group B (Table 7). We found that postoperative fall of haemoglobin in the TXA group was significantly less than in the non-TXA group (p = 0.0000), which is consistent with a study done by Sepah et al. [20].

Postoperatively, in group A, only four patients (26.7%) needed blood transfusion while 13 patients (86.7%) in group B needed blood transfusion; this shows that a significantly less number of patients required postoperative blood transfusion in group A (p = 0.0004) (Table 8).

In our study, in both groups A and B, there was no statistically significant difference in SGOT and SGPT, and this coincides with the results of a study done by Benoni et al. [21], which also shows that TXA does not alter liver functions (Tables 9, 10).

In our study, in both groups A and B, there was no significant difference in blood urea and serum creatinine (Table 11). These results are similar to a study done by Goswami et al. [22], which shows that TXA does not alter renal functions.

In our study, there was no statistically significant difference seen in bleeding time, clotting time, and PT/INR in either group; this shows that TXA has no adverse effect on these parameters (Table 12).

This study did have certain restrictions. First, this study had a small sample size of 30 patients. Second, the volume computed would undoubtedly be a little bit less than the real loss because the blood loss that we calculated in our study did not include the amount collected in the suction bottle during surgery. Almost all similar studies that we came across have excluded the loss in the suction bottle during TKR. During surgery, swabs were changed when felt like fully soaked. The number of blood-soaked swabs was considered a parameter for intraoperative blood loss; however, swabs were not weighed and this could be considered a limitation of the study. Furthermore, blood loss during surgery also depends on the duration of surgeries, which in turn also depends upon the skill and experience of the surgeons. Blood loss was significantly lower in the TXA group. This could imply that despite the extended surgery duration's higher risk of blood loss, TXA was able to lower it without noticeably increasing the risk of adverse events.

## Conclusions

The data from this study conclude that the use of TXA in TKR significantly reduces perioperative blood loss and reduces the need for blood transfusion in patients undergoing TKR without increasing any thromboembolic complications like DVT. Postoperatively, significantly less reduction in haemoglobin level and less requirement of blood transfusion was observed in the TXA group. TXA has no adverse effect on the liver and renal functions. Safety, low cost, ease of use, good bioavailability, high efficacy, and low complications have helped to increase the common use of TXA in TKR.
